# Plasma and Synovial Fluid Cell-Free DNA Concentrations Following Induction of Osteoarthritis in Horses

**DOI:** 10.3390/ani13061053

**Published:** 2023-03-14

**Authors:** Luca Panizzi, Keren E. Dittmer, Matthieu Vignes, Jennie S. Doucet, Kristene Gedye, Mark R. Waterland, Chris W. Rogers, Hiroki Sano, C. Wayne McIlwraith, Christopher B. Riley

**Affiliations:** 1 School of Veterinary Science, College of Science, Massey University, Palmerston North 4442, New Zealand; k.e.dittmer@massey.ac.nz (K.E.D.); k.gedye@massey.ac.nz (K.G.); c.w.rogers@massey.ac.nz (C.W.R.); h.sano@massey.ac.nz (H.S.); criley03@uoguelph.ca (C.B.R.); 2 School of Mathematical and Computational Sciences, College of Science, Massey University, Palmerston North 4442, New Zealand; m.vignes@massey.ac.nz; 3 Department of Biology, Faculty of Science, University of Waterloo, Waterloo, ON N2L 3G1, Canada; jennied.d1@gmail.com; 4 School of Natural Sciences, College of Science, Massey University, Palmerston North 4442, New Zealand; m.waterland@massey.ac.nz; 5 School of Agriculture and Environment, College of Science, Massey University, Palmerston North 4442, New Zealand; 6 Orthopaedic Research Center, C. Wayne McIlwraith Translational Medicine Institute, School of Veterinary Medicine, Colorado State University, Fort Collins, CO 80523-1601, USA; wayne.mcilwraith@colostate.edu; 7 Department of Clinical Sciences, Ontario Veterinary College, University of Guelph, Guelph, ON N1G 2W1, Canada

**Keywords:** carpus, cell-free DNA, horse, biomarker, osteoarthritis, plasma, synovial fluid

## Abstract

**Simple Summary:**

Biological molecules (biomarkers) have been investigated for the diagnosis of osteoarthritis, but their use in equine patients has been limited due to their high cost and limited accuracy and practicality. New biomarkers that address these problems may aid in the early diagnosis and treatment of equine osteoarthritis. This study aimed to determine if the measurement of cell-free DNA, which circulates outside of cells, could be used for the early diagnosis of experimentally created osteoarthritis in horses. Osteoarthritis was surgically induced in 9 of 17 healthy Thoroughbred fillies; the eight remaining horses served as a comparison group without osteoarthritis. Afterwards, joint fluid and plasma samples were collected weekly for 9 weeks, and cell-free DNA concentrations were measured. Biomarker concentrations were significantly higher for joint fluid from horses with osteoarthritis than the comparison group at 4 weeks (median 1430 μg/L and 631 μg/L, respectively) and 9 weeks (median 1537 μg/L and 606 μg/L, respectively). There were no significant differences in plasma cell-free DNA concentrations between the horse with early osteoarthritis and the comparison group. Plasma cell-free DNA measurement is not sufficiently sensitive for early diagnosis of osteoarthritis, while measurement of this biomarker in joint fluid may be useful.

**Abstract:**

Biomarkers for osteoarthritis (OA) in horses have been extensively investigated, but translation into clinical use has been limited due to cost, limited sensitivity, and practicality. Identifying novel biomarkers that overcome these limitations could facilitate early diagnosis and therapy. This study aimed to compare the concentrations of synovial fluid (SF) and plasma cell-free DNA (cfDNA) over time in control horses with those with induced carpal OA. Following an established model, unilateral carpal OA was induced in 9 of 17 healthy Thoroughbred fillies, while the remainder were sham-operated controls. Synovial fluid and plasma samples were obtained before induction of OA (Day 0) and weekly thereafter until Day 63, and cfDNA concentrations were determined using fluorometry. The SF cfDNA concentrations were significantly higher for OA joints than for sham-operated joints on Days 28 (median 1430 μg/L and 631 μg/L, respectively, *p* = 0.017) and 63 (median 1537 μg/L and 606 μg/L, respectively, *p* = 0.021). There were no significant differences in plasma cfDNA between the OA and the sham groups after induction of carpal OA. Plasma cfDNA measurement is not sufficiently sensitive for diagnostic purposes in this induced model of OA. Synovial fluid cfDNA measurement may be used as a biomarker to monitor early disease progression in horses with OA.

## 1. Introduction

Musculoskeletal injuries resulting in lameness are a leading cause of wastage in racing Thoroughbreds [1,2,3,4] and are associated with the highest number of training days lost [5]. Most lameness cases in the general horse population are associated with osteoarthritis (OA) [6,7,8]. The diagnosis of equine OA is commonly based on clinical evaluation supported by imaging (“dry biomarkers”) [9]. Unfortunately, imaging techniques have significant limitations in the early prediction and characterization of many musculoskeletal injuries and in providing sufficient data to inform prognostication. The primary constraints are a modest correlation between pain and imaging findings [10], low sensitivity [11] and specificity [12], and high cost for higher resolution modalities [13]. Extensive research on molecular biomarkers (“wet biomarkers”) for human OA during the last two decades [14,15] has paralleled progress in the characterization of equine orthopedic disease biomarkers [16,17]. Synovial fluid biomarkers have been used for horses in experimental settings to evaluate the health of cartilage and synovium using ELISA-based techniques [18,19,20,21,22]. However, these techniques have not translated into clinical use due to the tests’ lack of consistency, cost, and practicality of the tests [23]. Although serum biomarkers can identify horses with musculoskeletal injuries [24], the high cost of multiple ELISA techniques and limited sensitivity may prevent using these as screening tools for OA in a clinical setting. There is a need for noninvasive, repeatable diagnostic and screening tests with high sensitivity and specificity for early disease diagnosis, stratification of orthopedic disease, and the development and evaluation of new therapeutic approaches [9,25,26,27]. Cell-free DNA (cfDNA) consists of short sequences of double-stranded DNA that circulate in plasma in an unbound form [28]. The presence of unbound DNA in human serum has been known for over 70 years [29]. It has been found in increased concentrations in patients affected by myocardial infarction [30], lupus erythematosus [31], lymphoma, and other neoplastic conditions [32]. In the case of neoplasia, it is thought to derive from cell apoptosis and necrosis and has been associated with neutrophils in disease states [33]. Canine cfDNA has been measured and evaluated as a prognostic indicator of lymphoid neoplasia [34] for genomic aberration detection in mammary carcinomas [35] and for assessing the severity of acute pulmonary thromboembolism [36]. Equine fetal cfDNA in the maternal circulation has been used for fetal sexing [37,38]. Plasma cfDNA has been explored as a prognostic marker for septic and nonseptic illness in foals, but significant differences from healthy foals were not found [39]. In healthy subjects, circulating cfDNA originates predominantly from blood cells, endothelial cells, and hepatocytes, but in disease, the affected tissues also contribute to the circulating cfDNA [40,41]. Increased release of cfDNA into bodily fluids or decreased clearance may result in elevations or differences associated with disease, age, or sex [42,43]. Circulating cfDNA comprises sources from totally normal tissue sources, diseased tissues, and circulating mitochondrial DNA [44].

There is a growing interest in the role of cfDNA release from orthopedic tissues and responses in biofluid concentrations to disease and exercise [45,46,47]. An early report comparing human cfDNA values in synovial fluid and serum from patients with rheumatoid arthritis (RA), OA, and other orthopedic conditions found the highest concentrations in patients with RA compared to low levels in patients with OA or traumatic arthritis [48]. Further work exploring cfDNA in cases of RA has found that not only are elevations in the blood and synovial fluid indicators of disease, but they are also associated with disease progression [48]. Concentrations can also be monitored for other manifestations of immune-mediated joint disease [49]. Peer-reviewed publications investigating the diagnostic potential of cfDNA in equine orthopedic disease are lacking. Human plasma cfDNA concentration also increases with intense exercise [50,51,52,53,54,55]. For example, strenuous intermittent exercise increases plasma cfDNA in athletes [56,57]. Comparable published data for the horse are currently unavailable. 

The objectives of this study were to report the baseline concentrations of synovial fluid (SF) and plasma cfDNA in a group of young, healthy Thoroughbreds and to compare the concentrations of SF and plasma cfDNA over time in control horses with those with traumatically induced carpal osteoarthritis undergoing a standardized exercise regimen.

## 2. Materials and Methods

The study was approved by Massey University Animal Ethics Committee (protocol MUAEC 14/18). Fifteen 2-year-old and two 3-year-old New Zealand-bred Thoroughbred fillies (*n* = 17) that had not been used or trained for athletic activity were selected for the study. Inclusion criteria were lack of clinical abnormalities based on a daily general physical examination, lameness at the walk and trot, and absence of radiographic abnormalities of the carpi. Before the study, all horses were kept at pasture per normal New Zealand husbandry practice [58]. Before the trial and assignment to control and treatment groups, cfDNA was measured in plasma as described below from 16 of these horses during the same month (one (1/17) horse was not available until closer to the time of the experimental phase of the study). Based on the mean ± s.d. of these pretrial values (632 ± 91 μg/L), α = 0.05, and β = 0.20, a sample size for each group in the trial was estimated as *n* = 8. After blocking horses for sire (ensuring animals with the same sire were not within the same group) and age, each horse was assigned to one of two groups. For nine horses, an 8 mm osteochondral fragment was created arthroscopically in the distal dorsal aspect of the radial carpal bone of one randomly selected limb to induce OA using an established equine model [59,60]. Per the published protocol, the radial carpal bone fragment was left attached to the dorsal joint capsule reflection. The parent bone was debrided with a motorized burr to make a ~15 mm-wide defect (including the fragment’s width), and the debris was left in the joint as previously described. These horses were identified as the OA horse group, their operated middle carpal joints as OA joints, and the unoperated contralateral middle carpal joint as OA-control joints. The other eight horses (sham horse group) underwent arthroscopic exploration only of one randomly selected middle carpal joint (sham joint), and the unoperated contralateral middle carpal joint served as a sham-control joint. All horses were administered procaine penicillin (22 mg/kg, IM) once before surgery and phenylbutazone immediately after completion of the procedure (4.4 mg/kg, IV) and for the following 4 days (4.4 mg/kg, PO, q 24 h). During the trial, horses were housed in stalls with limited free exercise (30 min/day in a 6 m × 6 m yard) throughout the study. After a 14-day postsurgical recovery period, all horses started a 7-week-long treadmill exercise protocol (5 consecutive days, Monday through Friday each week, followed by 2 days of rest). On each treadmill day, horses were exercised for 2 min at a trot (4–5 m/s), followed by 2 min at a gallop (8–9 m/s), and then a further 2 min at a trot (4–5 m/s). The model has been reported to mimic naturally occurring equine traumatic OA [61]. Horses were assessed and scored for lameness at the walk and trot, joint effusion, and response to carpal flexion preoperatively and weekly thereafter by the same board-certified surgeon. Lameness scores were assigned according to the American Association of Equine Practitioners lameness scale [62]. Radiographs of both carpi were obtained for all horses before entering the study and at its completion. For these images, scores were assigned by two board-certified radiologists blinded to group allocation based on lysis and osteophyte formation. These clinical and radiographic scores confirming the establishment of OA in this study have been published [63]. Blood (~10 mL) was collected by venipuncture and SF (~3–4 mL) via bilateral middle carpal arthrocentesis from all horses starting on Day 0, immediately before induction of OA or sham surgery and weekly each Monday before the commencement of exercise until Day 63. Blood samples were chilled on ice, and approximately 4–5 mL of plasma was obtained after centrifugation within 60 min of collection. The plasma obtained was divided into 1 mL aliquots and stored at −80 °C for later analysis. An aliquot (~0.5 mL) of SF was placed in ethylenediaminetetraacetic acid (EDTA) tubes and processed to determine the total nucleated cell count (TNCC) and nucleated cell differential count. A refractometer determined the total protein concentration on fresh SF immediately after collection. The remainder of the SF was divided into ~1 mL aliquots and stored at −80 °C until later analysis. At the time of cfDNA analysis, plasma and SF samples were thawed at room temperature and divided into aliquots of 20 μL. cfDNA concentrations were measured by fluorometry (Qubit dsDNA High Sensitivity Assay kit, Life Technologies, Carlsbad, CA, USA) in triplicate using a fluorometer (Qubit 2.0 fluorometer, Life Technologies, Carlsbad, CA, USA) according to the manufacturer’s specifications [64]. Calibration was performed with the standards provided by the manufacturer before each run. The fluorescence detected by the fluorometer is proportional to the concentration of double-strand DNA in the sample. For each plasma sample, cfDNA replicate values were averaged. Plasma cfDNA data were not normally distributed and were analyzed with Freidman’s two-way ANOVA to account for repeated measures (Statistica 11, StatSoft, Tulsa, OK, USA). When significant differences were observed, post hoc analysis was performed using Wilcoxon matched-pairs test to detect the sources of temporal differences within OA and control groups. Synovial fluid data were not normally distributed, and outliers were eliminated (excluded if a value outside of the range of [Q1 − 1.5 x IQR, Q3 + 1.5 x IQR]) at each time point. For each SF sample, cfDNA replicate values were averaged. A paired comparison of SF cfDNA between left and right joints on Day 0 was performed using Kruskal–Wallis ANOVA. Synovial fluid from OA joints was compared with SF from OA-control joints and SF from sham joints over time using Freidman’s two-way ANOVA. Where differences were significant, post hoc analysis using Wilcoxon matched-pairs test and Mann–Whitney U tests was performed to detect the sources of temporal differences within groups. Lameness, flexion tests, effusion, radiographic scores, and TNCC were analyzed using Freidman’s two-way ANOVA. Where differences were significant, post hoc analysis using Mann–Whitney U tests was performed. Similarly, Freidman’s two-way ANOVA and Wilcoxon matched-pairs test were used for total protein concentrations. Significance was set at *p* < 0.05. The precision of the assay in SF and plasma was determined for these samples. Triplicate fluorimetry measurements of each sample were averaged, and the coefficient of variation (CV) was calculated using the standard deviation (s.d.) and mean (μ) of the replicates for each sample (CV = s.d./μ) [65].

## 3. Results

### 3.1. Synovial Fluid cfDNA

**Figure 1:**
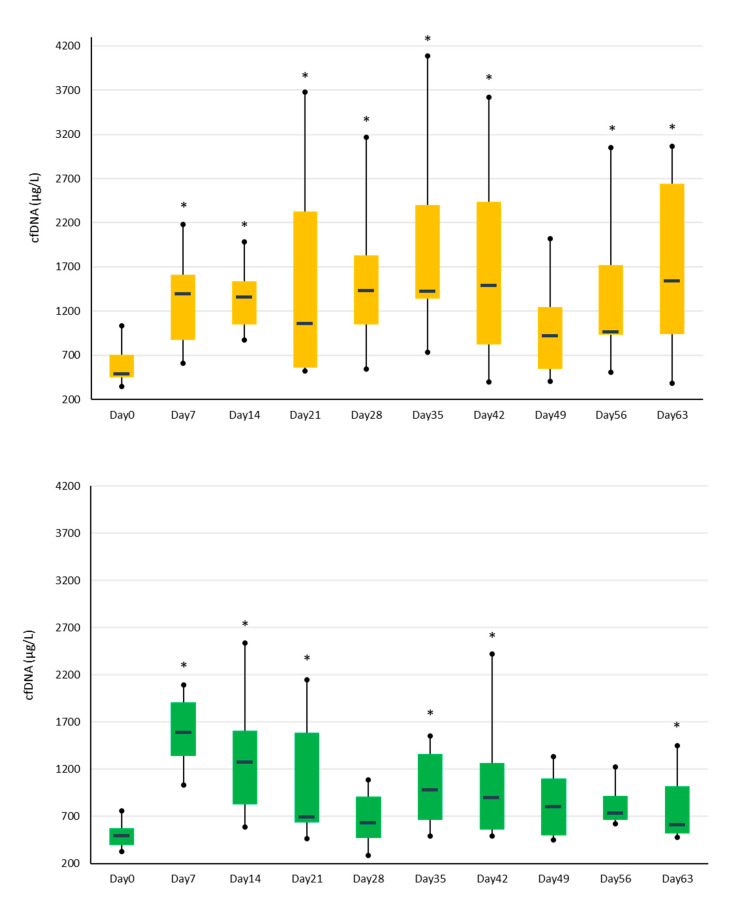
Synovial fluid cell-free DNA concentrations in the OA (top graph) and sham (bottom graph) joints over time. The * indicates significant differences (*p* < 0.05) from the respective Day 0 values. The bars represent the interquartile range, and the horizontal lines within the bars are the median values. Whiskers indicate the minimum and maximum values.

On Day 0, the baseline median SF cfDNA concentration was 504 μg/L (IQR = 236), and there was no significant difference between groups and between carpi within horses. After the creation of the osteochondral fragment, cfDNA concentrations in SF were significantly higher for OA joints than for sham-operated joints on Days 28 (median 1430 and 631, respectively, *p* = 0.017) and 63 (median 1537 and 606, respectively, *p* = 0.021); SF concentrations were trending towards significance for OA joints on Days 35 (*p* = 0.059) and 56 (*p* = 0.070). Within OA horses, there was a significant difference in concentrations over time in the OA joints (*p* = 0.036) but not in the OA controls (*p* = 0.12). SF cfDNA concentrations for OA joints were significantly higher at all time points (*p* range: 0.010– 0.043) compared to Day 0, except for Day 49 (Figure 1).

### 3.2. Plasma cfDNA

**Figure 2:**
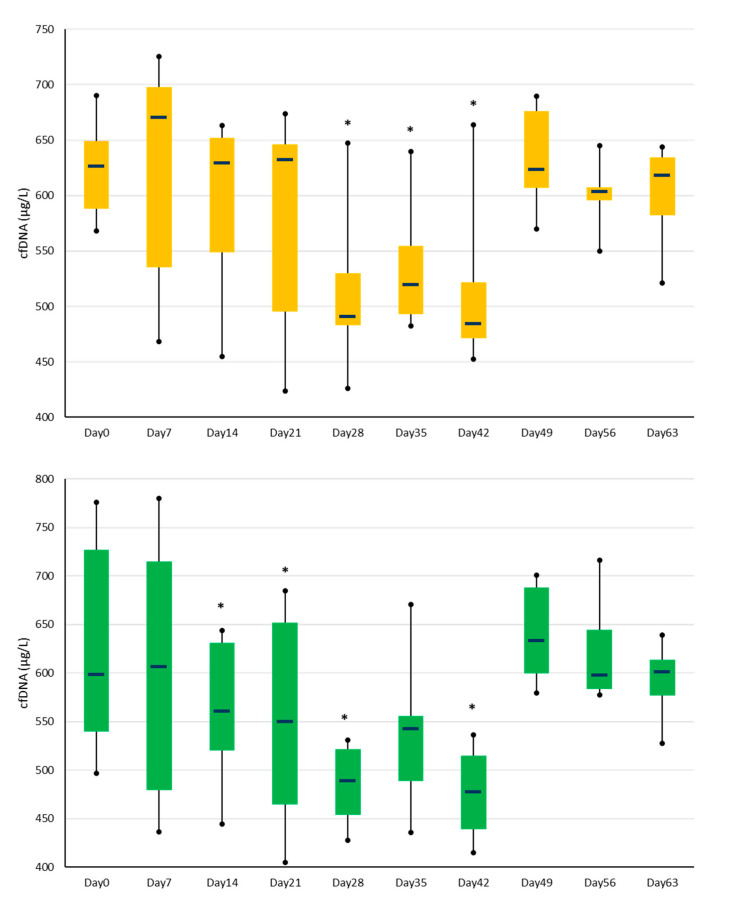
Plasma cell-free DNA concentrations in the OA (top graph) and sham (bottom chart) groups over time. The * indicates significant differences (*p* < 0.05) from the respective group Day 0 values. The bars represent the interquartile range, and the horizontal lines within the bars are the median values. Whiskers indicate the minimum and maximum values.

The baseline median plasma cfDNA concentration on Day 0 was 626 μg/L (IQR 117), and there was no significant difference between groups. No statistically significant effect of the OA model on plasma cfDNA concentrations was detected. However, significant differences in plasma cfDNA concentrations over time were identified. This was true for pooled data (all horses), where Day 0 values significantly differed from those at Days 14, 21, 28, 35, and 42. This was also confirmed within groups: In the OA group, Day 0 concentrations were significantly higher than those for Days 28, 35, and 42 (*p* < 0.05); in the sham group, Day 0 values were significantly higher than those for Days 14, 21, 28, and 42 (*p* < 0.05) as shown in Figure 2.

### 3.3. Clinical Parameters

Synovial fluid TNCCs did not differ significantly between groups on Day 0 and over time within groups. There were no significant TNCC differences between OA and sham joints, OA and OA-control joints, sham and sham-control joints, and OA-control and sham-control joints. Total protein concentrations in SF were not significantly different between operated sham joints and OA joints at Day 0 or any other time throughout the study, except on Day 14 (*p* = 0.01). No significant differences were observed between sham-control and OA-control joints.

### 3.4. Assay Precision

The precision of the assay for cfDNA was high. The mean plasma coefficient of variation (CV) was 2.70% (95% CI 2.45–2.96), and almost all CV values fell within 5% of the mean CV. The synovial fluid mean CV was 0.95 % (95% CI 0.87–1.03), and almost all CV values fell within 3% of the mean CV.

## 4. Discussion

To the authors’ knowledge, this study is the first to report cfDNA concentrations in SF and plasma of healthy young racehorses. An osteochondral fragment model of carpal OA was used to investigate the use of cfDNA as a potential biomarker for OA. Our results show that, although the concentration of cfDNA in plasma was not useful for differentiating horses affected by induced carpal OA from controls, its use in the synovial fluid has potential value for monitoring change over time.

The SF of both OA and sham joints contained significantly increased cfDNA concentrations compared to the respective Day 0 values. The increase in sham joints may be due to the effect of multiple arthrocentesis during the study. However, although multiple arthrocentesis have been shown to induce an increase in OA biomarkers in SF of exercised horses, this effect is minimized when weekly intervals between arthrocentesis are used, as was completed in our study [66]. The early increase in cfDNA concentration in sham joints may be attributed to the traumatic effect of the sham surgery. Similar observations have been made in sham joints with significant increases in the anti-inflammatory mediator interleukin-1 receptor antagonist and the pro-inflammatory cytokine tumor necrosis factor alpha after arthroscopy [67].

Compared with sham joints, the significant change over time of SF cfDNA concentrations in OA joints suggests that the effects of induced OA were sustained, and that exercise was not responsible for the observed differences. This is consistent with previous research where the effects of exercise on synovial fluid biomarkers differed from those caused by the induction of OA [16]. Although the half-life of cfDNA in horses has not been documented, it is likely to be within the range reported in people and dogs (1.5 h and 5.6 h, respectively) [68,69]. In the current study, synovial fluid samples were taken weekly approximately 72 h after cessation of the previous week’s exercise. The authors, therefore, suggest that any increase in the concentration of cfDNA is unlikely to be associated with exercise but rather with the ongoing contributions from the diseased tissues.

To the authors’ knowledge, there is no peer-reviewed published literature on cfDNA in SF in horses. The significance of the current findings concerning naturally occurring OA remains unknown at this stage. When compared with sham joints, the significantly higher concentrations of SF cfDNA at Days 28 and 63 in OA joints confirm that SF cfDNA measurement can identify OA-affected joints in a research setting. However, validation in cases with naturally occurring disease is necessary before making recommendations for its clinical use.

The current study did not show a significant difference between plasma cfDNA concentrations of horses with induced OA of the middle carpal joint and horses of the sham group over 2 months. Median plasma cfDNA concentrations in the OA group were higher than in the sham group at most time points. However, because significant differences in this biomarker between OA and sham horses were not found for plasma cfDNA, the authors conclude that it is not a sensitive predictor of experimental OA for this biofluid. An ideal property of plasma biomarkers is a high level of sensitivity in detecting disease. This means that the molecules being detected, in this case, cfDNA, must be circulating in sufficient concentrations associated with the burden of disease to be detected. Studies exploring the role of cfDNA in people and dogs have investigated more severe conditions (cancer, severe trauma, sepsis, rheumatoid arthritis, and acute pulmonary thromboembolism), with significant systemic responses and a high burden of disease [36,45,46,47]. Frisbie et al. [16] reported significant changes in serum biomarkers of cartilage degradation using the same OA model. However, as observed for other species with different medical conditions, a more substantial or naturally occurring disease burden may be required to generate significant increases of OA-associated cfDNA concentrations in the peripheral circulation [36,45,46,47]. Even though, a similar lack of diagnostic sensitivity was demonstrated in a recent study in foals that showed no significant differences in cfDNA concentrations between critically ill and healthy foals [39], suggesting that even with significant systemic illness, plasma cfDNA concentration may not be a useful marker in young horses. In contrast, cfDNA is significantly higher in emergency patients > 2 years old with colic than in control horses and ponies [70]. In the current OA model, plasma cfDNA measurement was not sufficiently sensitive for diagnostic purposes.

Nevertheless, temporal changes in plasma cfDNA concentrations were observed in both groups at Days 14 and 28 after surgery in the sham and OA groups, respectively. Thereafter, a decline in cfDNA concentrations was seen until 4 weeks after the start of training, after which cfDNA concentrations returned to baselines. The reason for this is unknown, as there is limited information on the effects in vivo of physical loading on the integrity and metabolism of synovium [71]. In humans, plasma cfDNA concentrations increase with exercise but rapidly decrease within 30 min of cessation of exercise [53], returning to baseline values within 2 h [52]. Similarly, in working dogs, exercise is associated with a transient increase in cfDNA concentrations 30 min after the end of the exercise, returning to baseline levels 90 min thereafter [72]. Details of the effects of the training regime on cfDNA concentrations in the horses in the current study are unknown, but further investigation of the half-life cfDNA in equids and the optimal time points for sampling after exercise or disease induction is recommended.

Based on the current results, plasma cfDNA concentrations alone do not provide significant information for focal diseases such as that evaluated in this study. In the future, it may be helpful to investigate whether specific sequences within the cfDNA rather than total cfDNA concentrations, as described for specific human cancers [73,74], better identify horses affected by OA.

The small CV found for the cfDNA assay in horses is consistent with the mean CV (2%) using the same analytical method reported in dogs [75] and a report that used the assay in horses admitted as emergency patients with colic and a control group [70]. The precision of the assay meets the USA Food and Drug Administration’s requirements for bioanalytical method validation (Food and Drug Administration, 2018). The precision of the assay supports further use of this fluorometric method for future studies of equine conditions with a high burden of disease.

The TNCC in SF did not change significantly within and between groups over time. Arthrocentesis causes an increase in SF TNCC [76], although transient (1–2 days), which may be a confounding factor when utilizing TNCC as an outcome measure. Since both groups were sampled during the same timeframe and with equal frequency, small differences in TNCC may have been skewed by the response to arthrocentesis. However, the effects of repeated arthrocentesis on TNCC are generally short-lived and not likely to be a factor in our study, given the 7-day interval between samplings. Total protein concentrations in SF did not significantly change over time and among groups. The lack of significant increases in SF TNCC and total protein concentration in our study is consistent with mild to no increase generally observed in joints affected by OA [77], but in contrast with other studies in which this same carpal OA model was used [16]. The reasons for the differences between the work of Frisbie et al. [16] and the current study are unclear as the technique used for OA model was under the direction of an author common to both studies, and both reports used 2-year-old horses. To reduce interhorse variation as a random source of error, the current study used a cohort of the same breed and gender, bred, and raised at pasture in the same country. These signalment details were not described in the report by Frisbie et al. [16]. Such differences, if present, may have contributed to SF TNCC findings in the current study that were not consistent with those from the previously published work.

### Study Limitations

The lack of an age-matched unexercised control group limited the interpretation of the role of exercise on cfDNA concentrations. However, based on the short half-life of cfDNA reported in other species, the timing of sampling 72 h after treadmill exercise is likely to have prevented the detection of confounding exercise-related effects in the current study. Determination of the half-life of this marker in horses may facilitate the identification of the optimal time for sampling.

To reduce variably in results attributed to these factors, differences due to age or gender were not explored in the current study. Plasma cfDNA concentrations are higher in older (20 months) rats compared to younger ones (3 months) [78]. In humans, individuals over 60 [79] and 90 years of age [80] have higher concentrations of cfDNA. Gender has not been associated with differences in human cfDNA concentration except in women over 60 who had higher concentrations [79]. A larger equine population with different age categories, inclusive of intact and castrated males, may be more representative of establishing baseline concentration ranges for cfDNA in the wider population of horses. 

## 5. Conclusions

This study reports baseline plasma and SF cfDNA concentrations in horses and examines the effects of focal orthopedic trauma and exercise on this marker. The OA induced using a well-established model in the middle carpal joint in horses did not affect plasma cfDNA concentrations over 2 months suggesting this biomarker is not a valuable tool for early detection using this biofluid. Conversely, SF cfDNA concentrations have the potential to detect OA in an experimental setting and with further evaluation may be suitable for monitoring disease progression. Further study and clinical validation are necessary before recommending its use in the field for equine practice. 

## Data Availability

The data presented in this study are available on request from the corresponding author.
